# Characteristics of Patients with End-Stage Kidney Disease in ADPKD

**DOI:** 10.1016/j.ekir.2020.12.016

**Published:** 2020-12-31

**Authors:** Shehbaz S. Shukoor, Lisa E. Vaughan, Marie E. Edwards, Sravanthi Lavu, Timothy L. Kline, Sarah R. Senum, Yaman Mkhaimer, Ghaith Zaatari, Maria V. Irazabal, Reem Neal, Marie C. Hogan, Ziad M. Zoghby, Peter C. Harris, Vicente E. Torres, Fouad T. Chebib

**Affiliations:** 1 Division of Nephrology and Hypertension, Mayo Clinic College of Medicine, Rochester, Minnesota, USA; 2 Division of Biostatistics, Mayo Clinic College of Medicine, Rochester, Minnesota, USA; 3 Division of Radiology, Mayo Clinic College of Medicine, Rochester, Minnesota, USA

**Keywords:** ADPKD, Aging, Characteristics of ESKD in ADPKD, ESKD, Gender, Polycystic Kidney Disease, TKV, Total Kidney Volume

## Abstract

**Introduction:**

Cystic expansion damaging the parenchyma is thought to lead to end-stage kidney disease (ESKD) in autosomal dominant polycystic kidney disease (ADPKD). Here we characterized genotypic and phenotypic attributes of ADPKD at time of ESKD.

**Methods:**

This is a retrospective cross-sectional study of patients with ADPKD with ESKD evaluated at Mayo Clinic with available abdominal computed tomography (CT) or magnetic resonance imaging (MRI). Kidney volumes were measured (total kidney volume adjusted for height [HtTKV]), Mayo Image Class (MIC) calculated, ADPKD genotype determined, and clinical and laboratory features obtained from medical records.

**Results:**

Differences in HtTKV at ESKD were associated with patient age and sex; older patients and women had smaller HtTKV at ESKD. HtTKV at ESKD was observed to be 12.3% smaller with each decade of age (*P* < 0.01); but significant only in women (17.8%, *P* < 0.01; men 6.9%, *P* = 0.06). Patients with onset of ESKD at <47, 47–61, or >61 years had different characteristics, with a shift from youngest to oldest in male to female enrichment, MIC from 1D/1E to 1B/1C, likely fully penetrant *PKD1* mutations from 95% to 42%, and presence of macrovascular disease from 8% to 40%. Macrovascular disease was associated with smaller kidneys in female patients.

**Conclusion:**

HtTKV at ESKD was smaller with advancing age in patients with ADPKD, particularly in women. These novel findings provide insight into possible underlying mechanisms leading to ESKD, which differ between younger and older individuals. Cystic growth is the predominant mechanism in younger patients with ESKD, whereas aging-related factors, including vascular disease, becomes potentially important as patients age.

## Graphical abstract

**Figure:**
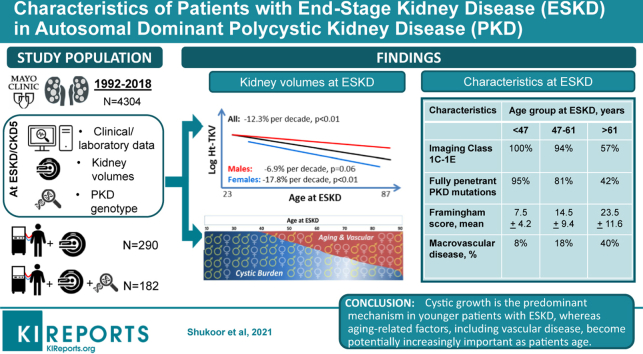

ADPKD is the leading genetic and the fourth overall cause of ESKD.^1^ It is mainly caused by mutations to *PKD1* or *PKD2*. Patients with *PKD1* mutations have more severe disease than those with *PKD2* mutations, reaching ESKD ∼20 years earlier.^2^ The type of *PKD1* mutation also affects prognosis; patients with *PKD1* truncating (*PKD1*^T^), *PKD1* nontruncating (*PKD1*^NT^), and *PKD2* mutations reach ESKD at 55, 67, and 79 years of age, respectively.^3^ More recently, *PKD1* missense mutations have been subdivided into likely fully penetrant (*PKD1*^NT1^) or hypomorphic (*PKD1*^NT2^).^4^^,^^5^ The severity of *PKD1*^NT1^ disease is similar to that of *PKD1*^T^, whereas the severity of *PKD1*^NT2^ disease is closer to *PKD2*.^5^

Much of the current understanding of the natural history of ADPKD derives from the Consortium for Radiologic Imaging Studies of Polycystic Kidney Disease (CRISP), an observational study now in its 19th year.^6^, ^7^, ^8^ In this study, patients with *PKD1* had larger kidneys and more cysts than patients with *PKD2*, but the rate of growth of the kidneys was not significantly different, suggesting that the main difference between *PKD1-* and *PKD2-*associated disease is the number of cysts developing at a relatively early stage of disease, rather than their rate of growth.^9^ CRISP also demonstrated that TKV (total kidney volume) adjusted for height (HtTKV) is a powerful predictor of renal function decline.^6^ These results provided the basis for larger studies^10^^,^^11^ that led to the approval of TKV as a prognostic biomarker, by both the US Food and Drug Administration^12^ and the European Medicines Agency,^13^ and for the development of the ADPKD HtTKV/age MICs.^14^ In addition to TKV, CRISP has also shown that renal blood flow is an independent predictor of renal function decline.^15^ However, the measurement of kidney blood flow is more challenging than that of TKV and thus a major limitation to its use. It has been proposed that cyst development and enlargement cause renal function decline via obstruction of tubular flow, release of cytokines and chemokines, and induction of interstitial inflammation and fibrosis.^16^ Given the proposed role of cystic enlargement as the primary cause of renal functional decline in ADPKD, we reasoned that HtTKV should be relatively similar in all patients at the time they reach ESKD regardless of the causative gene/mutation, age, and other environmental or clinical factors. Therefore, the purpose of our study was to characterize genotypic and phenotypic attributes of ADPKD at the time of ESKD, as well as compare the role of these factors relative to genotype and renal cystic expansion.

## Methods

### Study Patients

This is a retrospective cross-sectional study that includes Mayo Clinic (Minnesota, Florida, and Arizona) patients with ADPKD from January 1992 to January 2018, with data available when they reached ESKD or chronic kidney disease (CKD) Stage 5. Patients included in this study required all of the following: (i) diagnosis of ADPKD based on the Ravine-Pei modified criteria, (ii) diagnosis of ESKD or CKD Stage 5, and (iii) available abdominal imaging (CT/MRI) at the time of ESKD (<24 months before or <3 months after) (*n* = 290). The median time between the imaging and ESKD dates was 4.8 months before ESKD (interquartile range 0.7–11 months). Almost two-thirds (61.7%) of the patients are from Minnesota and surrounding states (Wisconsin, Iowa, South Dakota, and North Dakota). Patients were excluded if they had 1 or more of the following: (i) absence of electronic medical records, (ii) estimated glomerular filtration rate (eGFR) >15 ml/min per 1.73 m^2^ unless the patient received a preemptive kidney transplantation, (iii) presence of concomitant renal disease with major contribution to GFR decline, and (iv) procedures that affected TKV such as nephrectomy or cyst fenestration.

### Data Collection

Clinical data at the time of ESKD were carefully retrieved from the medical records. The date of ESKD was defined as first day of (i) preemptive transplantation, (ii) permanent dialysis, or (iii) when GFR was ≤15 ml/min per 1.73 m^2^, whichever occurred first. The retrieved data included age at ESKD, sex, race, height, history of hypertension, body mass index, lipid profile, smoking history, history of dyslipidemia, and history of macrovascular disease, as defined later in this article. Framingham risk scores were calculated using the Framingham calculator from the Framingham Heart Study.^17^ This scoring estimates the % risk of an adverse cardiovascular event in the next 10 years. The Framingham scores were calculated only for patients between 30 and 74 years of age, without prior history of cardiovascular disease. Factors included age, sex, systolic blood pressure, treatment for hypertension, currently smoking, diabetes, and body mass index. The eGFR was calculated using the CKD–Epidemiology Collaboration (CKD-EPI) GFR formula.^18^ As the study spans over 26 years, the serum creatinine of patients had been obtained through various standardization methods. The calibration error related to nonstandardized creatinine measurements is most important when eGFR is well preserved.^18^ This error has likely no effect on the estimation of eGFR by CKD-EPI formula in our study given that the patients included in this study have low eGFR.

Macrovascular disease was defined as 1 or more of the following before ESKD: (i) Stroke: ischemic or hemorrhagic; (ii) ischemic heart disease: (a) coronary artery disease: angina, intervention or (b) ischemic congestive heart failure: echocardiogram showing significant regional wall motion abnormalities; (iii) abdominal aortic aneurysm; and (iv) aortic calcification, which was graded for patients who had CT scans available (severe, moderate, mild, and none) based on extent of calcification observed in the descending aorta from the level of the diaphragm down to 3 cm below the aortic bifurcation. Only patients with severe or moderate grades were considered to have a diagnosis of aortic calcification. Patients with a history of ruptured intracranial aneurysm or intervention for intracranial aneurysm were also considered to have macrovascular disease.

Imaging near ESKD included CT or MRI of the abdomen within 24 months before ESKD date or up to 3 months after ESKD date. TKVs were calculated using planimetry or stereology and the HtTKV was calculated by dividing TKV by patient’s height. Although different acquisition sequences can introduce variability in measurement of TKV, this variability is comparable to the inter-reader differences and not likely to have affected the results. We have not used ultrasound measurements in our study because they are grossly inaccurate for very large kidneys, particularly in retrospective studies when only a few images are available. On the other hand, we have shown that MRI and CT produce comparable measurements of TKV.^14^ Mayo Class (1A through 1E and 2A-2B) was determined using the MIC calculator^14^ and categorized as detailed by Irazabal *et al.*^14^ Class 2 patients were divided into 2A (focal or asymmetrical) and 2B (atrophic kidneys) based on predetermined criteria listed in Irazabal *et al.*^14^ MIC (typical or class 1 vs. atypical or class 2) was determined by 2 adjudicators (SSS and FTC) and in unclear cases confirmed by a third adjudicator (VET).

A total of 778 patients were excluded because imaging satisfying our selection criteria was not available; 370 patients had no available CT/MRI imaging in our electronic systems and 408 patients had CT/MRI imaging but its timing was outside the designated imaging window. The lack of imaging in the first group was due to several factors: (i) some patients had only nonelectronic (hardcopy) imaging, (ii) electronic images before 1997 were not consistently archived, and (iii) some patients had only ultrasounds or presented to Mayo Clinic for non-nephrological care. Reasons accounting for the second group include (i) patients presented to Mayo Clinic after receiving a kidney transplant, or initiating dialysis, or (ii) had imaging studies outside the designated window and did not require a repeat imaging.

Based on the overall median age of ESKD, patients were stratified into 3 groups. Patients who reached ESKD at an age below the first quartile (Q1), between Q1 and Q3, and after Q3 were included in the first, second, and third group, respectively. The cutoff ages for Q1 and Q3 were rounded to the nearest number. In addition, patients were stratified by age group (5-year intervals) and sex for additional granularity. To assess for any temporal trends, patients were divided into tertiles based on the date of ESKD onset (1992–2000, 2001–2009, and 2010–2018).

### Genetic Analysis

The entire coding and flanking intronic regions of *PKD1* and *PKD2* were screened for mutations by Sanger or next generation sequencing as previously described.^19^, ^20^, ^21^, ^22^ Patients were classified as follows: *PKD1* truncating (*PKD1*^T^), *PKD1* nontruncating (*PKD1*^*NT*^), and *PKD2*. *PKD1*^NT^ mutations were subcategorized to *PKD1*^NT1 and^ *PKD1*^NT2^.^4^^,^^5^ *PKD1*^T^ and *PKD1*^NT1^ are defined as fully penetrant PKD mutations. A total of 185 of the 290 (63.8%) patients included in this study had genetic testing for *PKD1* or *PKD2* mutations. Among the 185 patients, 182 (98.4%) had *PKD1* or *PKD2* mutations and 3 (1.6%) had “no mutation detected.” Among the patients with no mutation detected, 1 patient had MIC 1E, 1 had MIC 1C, and 1 had MIC 2B.

### Statistical Analysis

Data were reported as mean ± SD for normally distributed data or median and interquartile range for skewed data, and *n* (%) for categorical data. *P*-values for comparisons by sex were derived using equal variance *t*-tests for data with normal distributions, Wilcoxon/Kruskal-Wallis tests for non-normal distributions, and χ^2^ tests for categorical variables. HtTKV was transformed using log base 10 to model an exponential growth process. Associations between HtTKV and patient characteristics were evaluated using both univariate and multivariate linear regression. The variables that were significantly predictive of HtTKV at ESKD at the 0.10 alpha level were included in the multivariate analysis. Beta coefficient estimates were derived from univariate or multivariate linear regression models using the log base 10 transformation on the outcome (HtTKV). The beta estimate was then transformed into percent change in HtTKV per unit increase in predictor variable. This percent change was calculated by subtracting 1 from the log of the estimate, then multiplying by 100 (% change/unit increase in predictor = [10^Beta Estimate^ – 1] × 100). Sensitivity analysis was performed on the patients with known genotype (*n* = 182) and on patients who reached ESKD in the latest period (2010–2018).

## Results

### Demographic and Clinical Characteristics at ESKD

**Figure 1:**
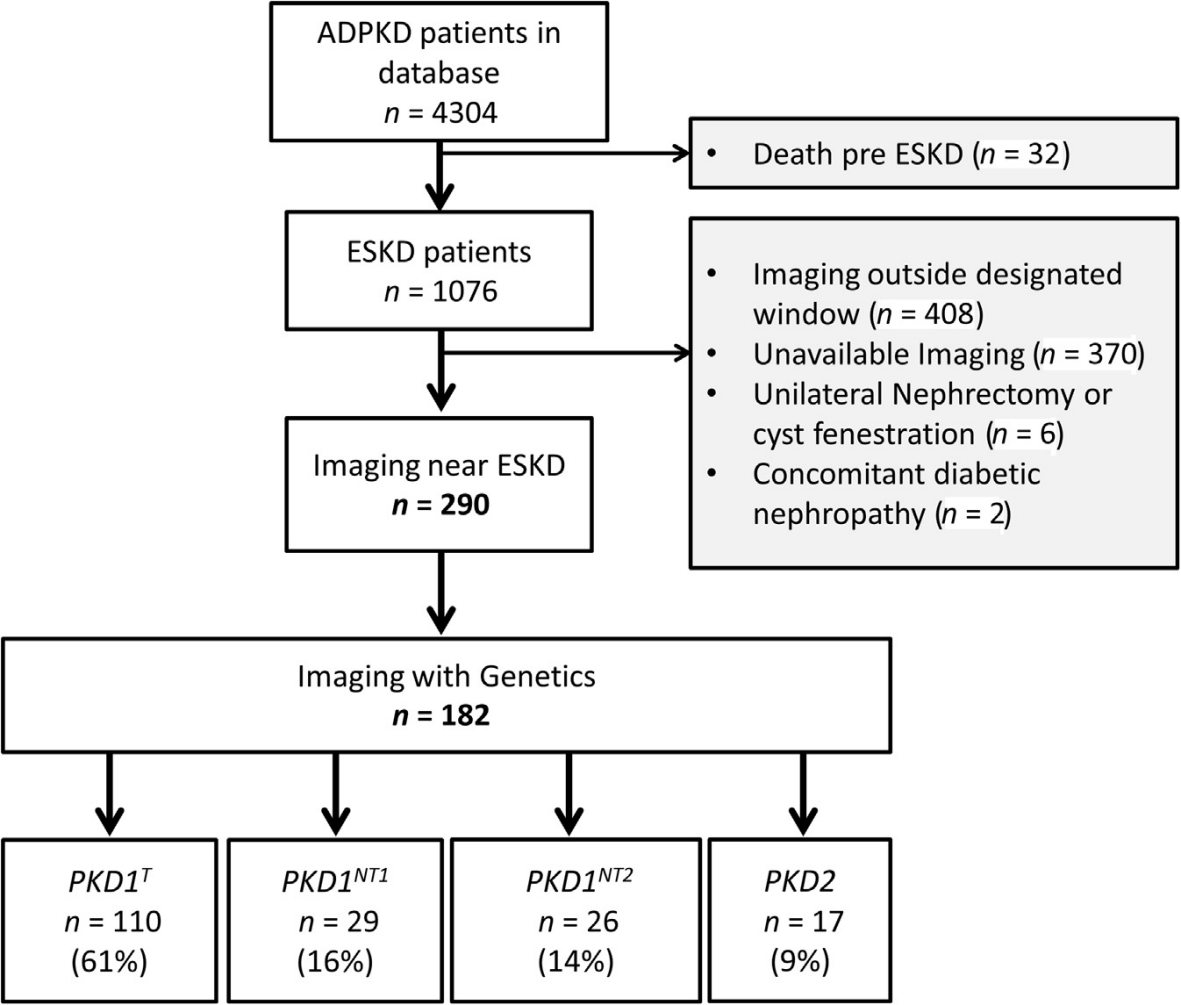
Study flow chart showing the number of patients who reached chronic kidney disease (CKD)5/end-stage kidney disease (ESKD), the number of patients with available kidney imaging, and the number of patients with available genetic classification.

The study flow chart is shown in Figure 1. Among 1076 patients with ADPKD who have reached CKD 5 or ESKD seen at Mayo Clinic between 1992 and 2018, 290 patients had abdominal imaging at time of ESKD. Among the included cohort, 179 patients had preemptive kidney transplantation (81 men, 98 women), 80 (48 men, 32 women) were receiving dialysis, and 31 (9 men, 22 women) were in CKD stage 5, but without real replacement therapy. The mean (± SD) eGFR was 14.1 (±5), 10.8 (±6), and 12.1 (±2) ml/min per 1.73 m^2^ in the preemptive kidney transplant, dialysis, and CKD stage 5 groups, respectively. The demographic and clinical characteristics of these patients, overall and separated by sex, are summarized in Table 1. The excluded patients showed similar demographics except for a lower proportion of preemptive kidney transplantation (37% vs. 62%) and higher body mass index (34 vs. 28.6 kg/m^2^) as compared with the included cohort (Supplementary Table S1). Men had higher HtTKV (mean: 2485 (±1263) ml/m versus 1611 (±1013) ml/m, *P* < 0.01) and higher MIC (Class 1C–E; 95% vs. 82%, *P* < 0.01) compared with women, despite similar mean ages at ESKD (54.2 and 54.8 years old, respectively) (Table 1). Men also had higher Framingham scores (mean: 18.9% (±11.6) vs. 10.7% (±7.5), *P* < 0.01) and were more likely to have a high-risk Framingham score (32% vs. 10%, *P* < 0.01) and evidence of macrovascular disease (26% vs. 16%, *P* = 0.04). Women, in contrast, had likely fully penetrant mutations more frequently than men (82% vs. 69%, *P* = 0.03). Body mass index and history of hypertension were similar in both sexes, whereas history of dyslipidemia and smoking were more frequent in men than women. Characteristics of patients who reached kidney failure before age 40 has been summarized in Supplementary Table S2.

**Table 1:** Demographic, clinical, and genotypic characteristics of all patients at ESKD separated by sex

Patient characteristic		All *N* = 290	Male *n =* 138 (48%)	Female *n =* 152 (52%)
**White, %y**		268 (92%)	129 (93%)	139 (91%)
**Age at ESKD (y), mean ± SD**		54.5 ± 11.3	54.2 ± 12.4	54.8 ± 10.3
**eGFR (ml/min per 1.73 m**^**2**^**), mean ± SD**				
All	*N* = 290	13.0 ± 4.6	13.1 ± 4.9	12.9 ± 4.2
Preemptive kidney transplant	*n =* 179	14.1 ± 4.6	14.4 ± 4.9	13.7 ± 4.3
Dialysis	*n =* 80	10.8 ± 4.6	10.8 ± 4.7	10.8 ± 4.6
CKD stage 5	*n =* 31	12.1 ± 1.8	12.5 ± 1.5	11.9 ± 2.0
**HtTKV (ml/m), median (interquartile range)**		1756 (1088–2614)	2315 (1458–3129)	1351 (872–2112)
**Mayo Image Class, *n***		**285**	**135**	**148**
1A		0	0	0
1B		30	5	25
1C		88	39	49
1D		90	41	49
1E		69	48	21
2B		8	2	6
**Severe class (1C–1E), %y**		247/285 (87%)	128/135 (95%)	122/148 (82%)
**Genotype, *n* (%)**				
PKD1^T^		110/182 (61)	42 (53)	68 (66)
PKD1^NT1^		29/182 (16)	13 (16)	16 (16)
PKD1^NT2^		26/182 (14)	12 (15)	14 (14)
PKD2		17/182 (9)	13 (16)	4 (4)
**Body mass index (kg/m**^**2**^**), mean ± SD**		28.6 ± 5.7	28.8 ± 5.2	28.5 ± 6.1
**History of hypertension, %y**		284 (98)	135(98)	149 (98*%*)
**History of smoking, %y**		122 (42)	66 (48)	56 (36*%*)
**History of dyslipidemia, %y**		133 (46)	72 (52)	61 (41*%*)
**Macrovascular disease, %y**		61 (21)	36 (26)	26 (16*%*)
**Framingham score characteristics**		***N* = 216**	***N* = 92**	***N* = 124**
Framingham score (%), mean ± SD		14.1 ± 10.3	18.9 ± 11.6	10.7 ± 7.5
High-risk Framingham score (≥20%), %y		42/216 (19*%*)	29/92 (32*%*)	13/124 (10*%*)

CKD, chronic kidney disease; eGFR, estimated glomerular filtration rate; ESKD, end-stage kidney disease; HtTKV, total kidney volume adjusted for height.

When stratified by quartile of age at ESKD, patients in the Q1 (<47 years) were more frequently men with an MIC of 1D or 1E, 95% had fully penetrant *PKD1* mutations, and 8% had macrovascular disease, whereas those in the Q2 and Q3 (47–61 years) were more often women, MIC 1C or 1D, 81% had fully penetrant *PKD1* mutations, and 18% had macrovascular disease. Patients in Q4 (>61 years) were also more often women, MIC 1B or 1C, only 42% had fully penetrant *PKD1* mutations, and 40% had macrovascular disease (Table 2). A more granular distribution of patients by age group (5-year interval) is shown in Supplementary Figure S1.

**Table 2:** Characteristics of patients divided by age quartiles when they reached ESKD

Patient characteristic	Age groups (at ESKD), y		
	Q1 (<47 y) *n* = 74	Q2+Q3 (47–61 y) *n* = 141	Q4 (>61 y) *n* = 75
**Male, *n* (%)**	**45 (61)**	**59 (42)**	**34 (45)**
**Mayo Imaging Class,***n***(%)**			
1E	49 (66)	19 (14)	1 (1)
1D	22 (30)	57 (40)	11 (16)
1C	3 (4)	57 (40)	28 (40)
1B	0 (0)	8 (6)	22 (32)
2B	0 (0)	0 (0)	8 (11)
**Mutation type or strength, *n* (%)**	***n* = 53**	***n* = 89**	***n* = 40**
PKD1^T^-PKD1^NT1^	55 (95)	72 (81)	17 (42)
PKD1^NT2^-PKD2	3 (5)	17 (19)	23 (58)
**Macrovascular disease, *n* (%)**	6 (8)	25 (18)	30 (40)
Framingham score,	***n* = 62**	***n* = 114**	***n* = 40**
% mean ± SD	7.5 ± 4.2	14.5 ± 9.4	23.5 ± 11.6

ESKD, end-stage kidney disease.

### Determinants of TKV at ESKD

**Figure 2:**
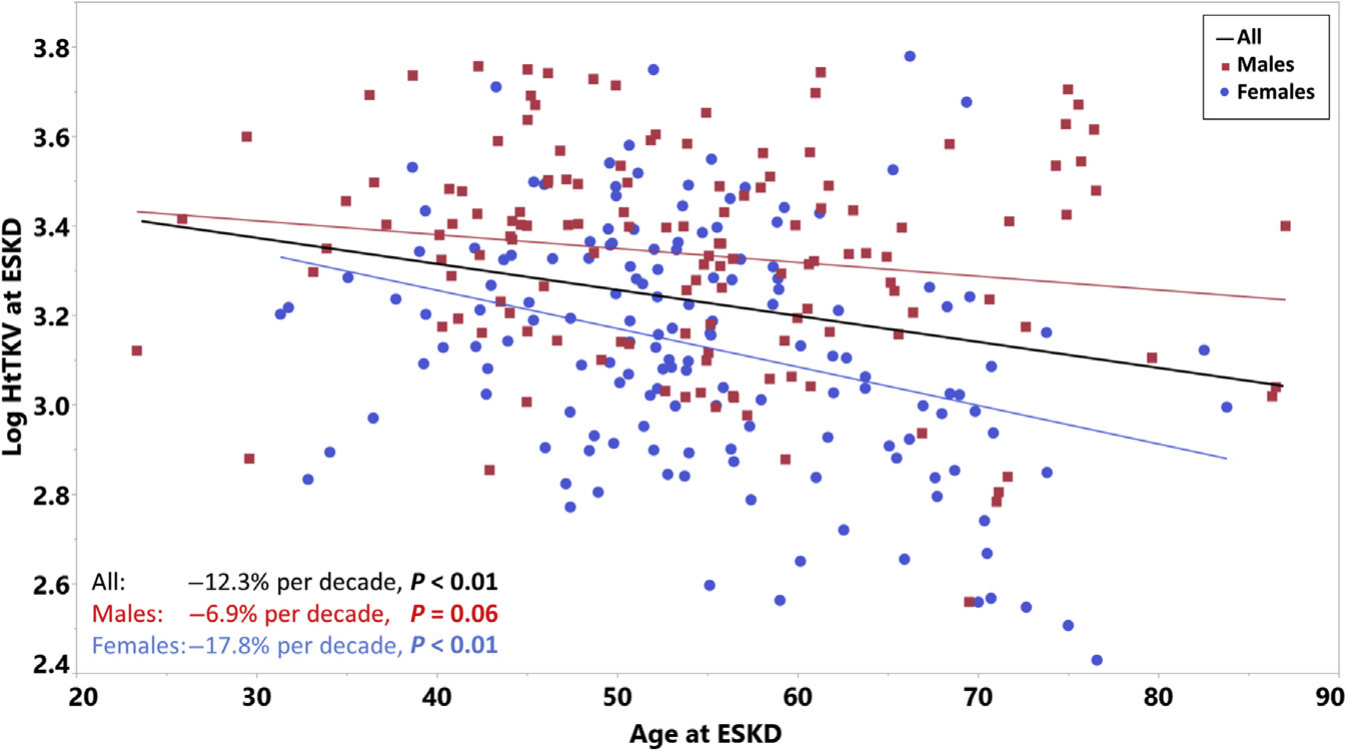
The Log10 total kidney volume adjusted for height (HtTKV) at end-stage kidney disease (ESKD) was plotted against the age at ESKD (*N* = 290). Best-fit lines and regression slopes are determined for all patients and by sex. The regression slope represents the percent change in HtTKV per decade of age at time of ESKD.

Interestingly, HtTKV at ESKD was found to be on average 12.3% smaller with each decade of age, *P* < 0.01; more in women (17.8% smaller per decade, *P* < 0.01) than in men (6.9% smaller per decade, *P* = 0.06) (Figure 2). Given the long period of patient recruitment (1992–2018), we also assessed whether there were any temporal trends in disease progression by dividing the cohort into 3 periods, 1992–2000 (*n* = 24), 2001–2009 (*n* = 100), and 2010–2018 (*n* = 166) (Supplementary Figure S2). Ages at ESKD and negative trends of HtTKV with age at time of ESKD were not significantly different across the 3 periods. The HtTKV negative trend per decade of age at ESKD was statistically significant for both sexes during the period 2010–2018, which was the period with the largest number of patients, but was attenuated during the earlier periods (*P* > 0.05 for both). In addition, we compared the trends of HtTKV with age at ESKD among patients who received preemptive kidney transplantation with those who received dialysis or had reached CKD stage 5. The overall trends for both groups were comparable (−11.9% vs. −13.3% smaller per decade of age in the kidney transplant group compared with the dialysis/CKD stage 5 group, respectively). When stratified by sex, HtTKV appeared smaller with age in men in the preemptive kidney transplant group compared with those in the dialysis/CKD stage 5 group (−12.1% vs. −4.9% per decade, respectively), whereas in contrast women tended to have smaller HtTKV with age in the dialysis/CKD stage 5 groups versus the kidney transplant group (−20.4% vs. −10.9% per decade, respectively) (Supplementary Figure S3).

Because of the large effect of sex on HtTKV across age at ESKD, we evaluated the association between other variables and HtTKV at ESKD stratified by men and women. Mutation severity and low risk Framingham score were associated with higher HtTKV at ESKD in men in the univariate analysis (Table 3, section A) but not in the multivariate analysis when age was included in the model (Table 3, section B). Age, Framingham score, and high-risk Framingham score were associated with lower HtTKV at ESKD in women in the univariate analysis (Table 4, section A), but only macrovascular disease was associated with lower HtTKV in the multivariate analysis when age was included in the model (Table 4, section B). Sensitivity analyses including only the patients with known genotype or the patients seen during the 2010–2018 period showed similar results (Supplementary Tables S3 and S4).

**Table 3:** Association between HtTKV at ESKD and clinical characteristics in male patients(univariate and multivariate analysis)

Predictor	*n*	Estimate^a^	95% CI	% change HtTKV^b^	*P* value
A - Univariate analysis- Male patients					
Age at ESKD (per decade)	138	−0.031	−0.062 to 0.001	−6.9	0.06
Genotype					
*PKD1*^*T*^*-PKD1*^*NT1*^	55	REF	REF	REF	REF
*PKD1*^*NT2*^*-PKD2*	25	−0.131	−0.242 to −0.019	−26.0	0.02
Body mass index (per 5 kg/m^2^)	134	0.025	−0.014 to 0.064	5.9	0.21
History of Smoking	138	0.040	−0.038 to 0.119	9.6	0.31
History of dyslipidemia	138	−0.060	0.139 to 0.018	−12.9	0.13
LDL (per 5 mg/dl)	50	−0.008	−0.018 to 0.001	−1.8	0.08
HDL (per 5 mg/dl)	47	−0.010	−0.034 to 0.013	−2.3	0.37
Framingham score (per 5%)	92	−0.017	−0.037 to 0.002	−3.8	0.07
High-risk score (≥20%)^c^	92	−0.113	−0.210 to −0.016	−22.9	0.02
Macrovascular disease	138	0.013	−0.077 to 0.103	3.0	0.77
B- Multivariate analysis- Male patients					
Age at ESKD (decades)	50	−0.061	−0.250 to 0.128	−13.1	0.52
Genotype	50				
*PKD1*^*T*^*-PKD1*^*NT1*^	-	REF	REF	REF	REF
*PKD1*^*NT2*^*-PKD2*	-	0.012	−0.208 to 0.231	2.8	0.92
LDL (per 5 mg/dl)	50	−0.001	−0.018 to 0.015	−0.2	0.87
Framingham Score (per 5%)	50	−0.015	−0.067 to 0.098	3.5	0.72
High-risk Framingham score^c^	50	−0.110	−0.496 to 0.277	−22.4	0.58

CI, confidence interval; ESKD, end-stage kidney disease; HDL, high-density lipoprotein; HtTKV, total kidney volume adjusted for height; LDL, low-density lipoprotein.

a Beta coefficient estimates derived from univariate linear or multivariate regression models using the log base 10 transformation on the outcome (HtTKV).

b Percent change in HtTKV per unit increase in predictor variable was calculated by subtracting 1 from the log of the estimate then multiplying by 100: % change/unit increase in predictor = (10^Beta Estimate^ −1) ×100.

c High-risk score is defined as having a Framingham score of ≥20%.

**Table 4:** Association between HtTKV at ESKD and clinical characteristics in female patients (univariate and multivariate analysis)

Predictor	*n*	Estimate^a^	95% CI	% change HtTKV^b^	*P* value
A- Univariate analysis- Female patients					
Age at ESKD (per decade)	152	−0.085	−0.124,−0.046	−17.8	<0.01
Genotype					
*PKD1*^*T*^*-PKD1*^*NT1*^	84	REF	REF	REF	REF
*PKD1*^*NT2*^*-PKD2*	18	−0.049	−0.172 to 0.072	−10.7	0.42
Body mass index (per 5 kg/m^2^)	148	0.025	−0.001 to 0.060	5.9	0.15
History of smoking	152	0.010	−0.077 to 0.098	2.3	0.05
History of dyslipidemia	150	−0.024	−0.110 to 0.061	−5.4	0.57
LDL (per 5 mg/dl)	56	−0.000	−0.009 to 0.009	0.0	0.99
HDL (per 5 mg/dl)	57	−0.017	−0.035,0.001	−3.8	0.06
Framingham score (per 5%)	124	−0.042	−0.071 to −0.012	−9.2	**<0.01**
High-risk score (≥20%)^c^	124	−0.155	−0.301 to −0.009	−30.0	**0.03**
Macrovascular disease	152	−0.103	−0.217 to 0.009	−21.1	0.07
B- Multivariate analysis- Female patients					
Age at ESKD (decades)	56	−0.020	−0.137 to 0.097	−4.5	0.74
History of smoking	56	−0.102	−0.255 to 0.051	−20.9	0.19
HDL (per 5 mg/dl)	56	0.003	−0.020 to 0.025	0.7	0.82
Framingham score (per 5%)	56	−0.031	−0.142 to 0.079	−6.9	0.58
High-risk Framingham score^c^	56	0.170	−0.243 to 0.583	47.9%	0.42
Macrovascular disease	56	-0.344	−0.674 to −0.014	−54.7%	**0.04**

CI, confidence interval; ESKD, end-stage kidney disease; HDL, high-density lipoprotein; HtTKV, total kidney volume adjusted for height; LDL, low-density lipoprotein.

a Beta coefficient estimates derived from univariate or multivariate linear regression models using the log base 10 transformation on the outcome (HtTKV).

b Percent change in HtTKV per unit increase in predictor variable was calculated by subtracting 1 from the log of the estimate then multiplying by 100: % change/unit increase in predictor = (10^Beta Estimate^ − 1) x 100.

c High-risk score is defined as having a Framingham score of ≥20%.

Of the initial cohort of 4304 patients in the ADPKD database, only 32 patients (0.7%) died without having ESKD, indicating that the selection bias in this study is minimal.

### Stratification of HtTKV at ESKD by MIC

**Figure 3:**
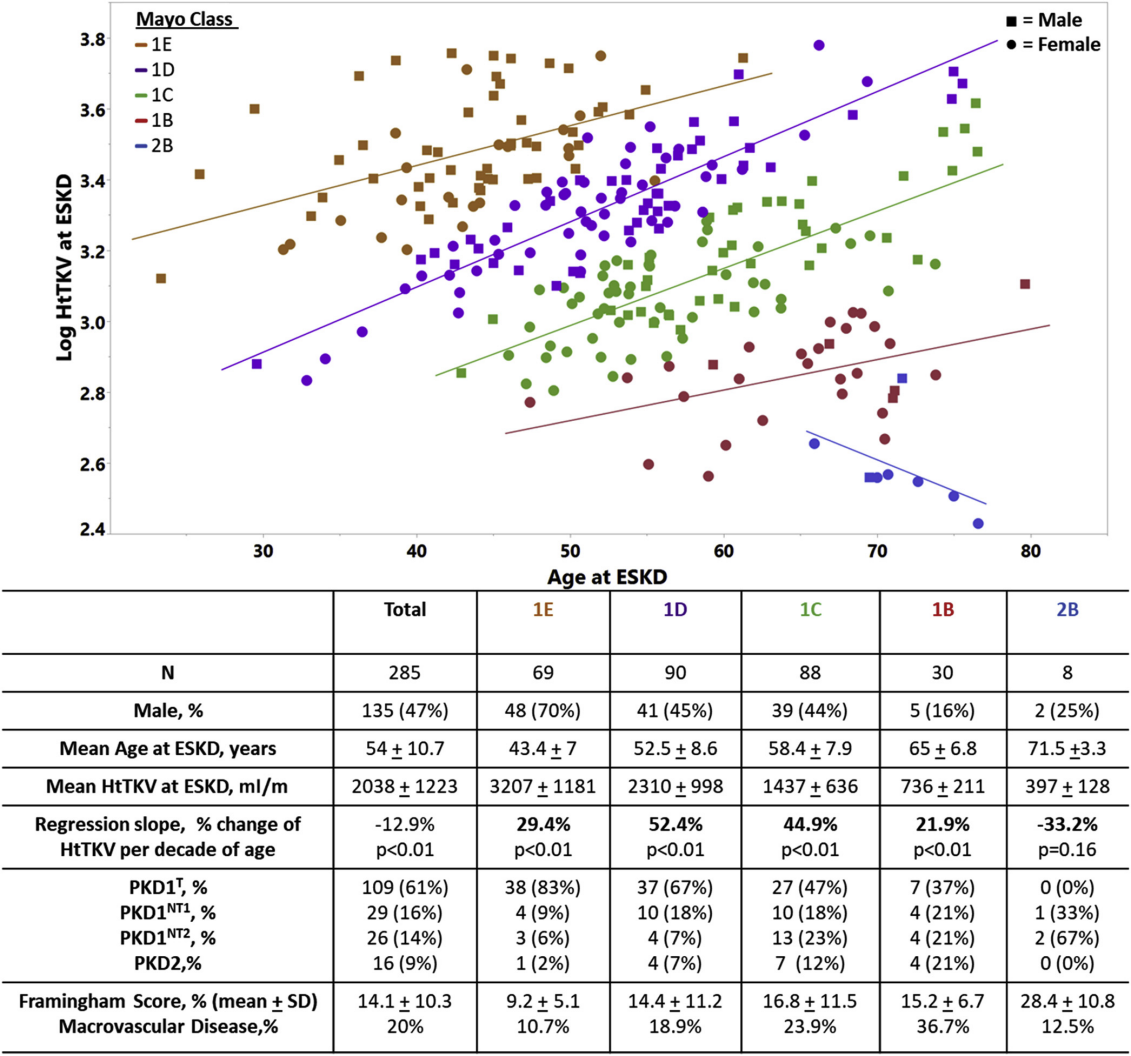
Log10 total kidney volume adjusted for height (HtTKV) at end-stage kidney disease (ESKD) was plotted against age at ESKD and stratified according to Mayo Imaging Class at the time of ESKD (*n* = 285). Best-fit lines and regression slopes are determined for each class. The regression slope represents the percent change in HtTKV per decade of age at time of ESKD. The table below the figure includes pertinent characteristics related to each specific Mayo Class.

MIC is calculated from HtTKV adjusted by age and is arguably the best currently available biomarker of disease severity.^14^^,^^23^ Because the MIC includes HtTKV in its derivation, we have excluded MICs from the analysis of variables associated with HtTKV at ESKD. To visually illustrate how disease severity and age relate to HtTKV at ESKD, we have plotted HtTKV and age stratified by MIC (Figure 3). Although overall HtTKV and age were negatively correlated, within each MIC1 group the correlation was positive, as expected given the criteria used to classify the patients with typical ADPKD. However, the correlation was less in class 1E patients than in class D and C patients who reached ESKD at a later age, likely reflecting the rapidity and severity of the cystic expansion associated with class 1E. On the other hand, the correlation was least in class 1B patients who reached ESKD at a much older age with likely contribution of age-related factors in addition to cystic expansion. Most patients (70%) in MIC 1E were men, whereas most patients (80%) in MIC 1B or 2B were women. The frequency of fully penetrant mutations decreased from 92% in MIC 1E to 58% in MIC 1B. Patients with ESKD with MIC1B (*n* = 30) were predominantly female (83%), with mean ESKD age of 65 (±6.8) years, with inadequately controlled hypertension (100%) and hyperlipidemia (57%); 27% had history of smoking and 37% had macrovascular disease. Patients with MIC 2B (*n* = 8) were predominantly women (75%), with mean ESKD age of 71.5 (±3.3) years, with inadequately controlled hypertension (62.5%) and hyperlipidemia (62.5%); 25% had history of smoking and 12.5% had severe aortic calcification.

### Phenotypic Variability Among Family Members

**Figure 4:**
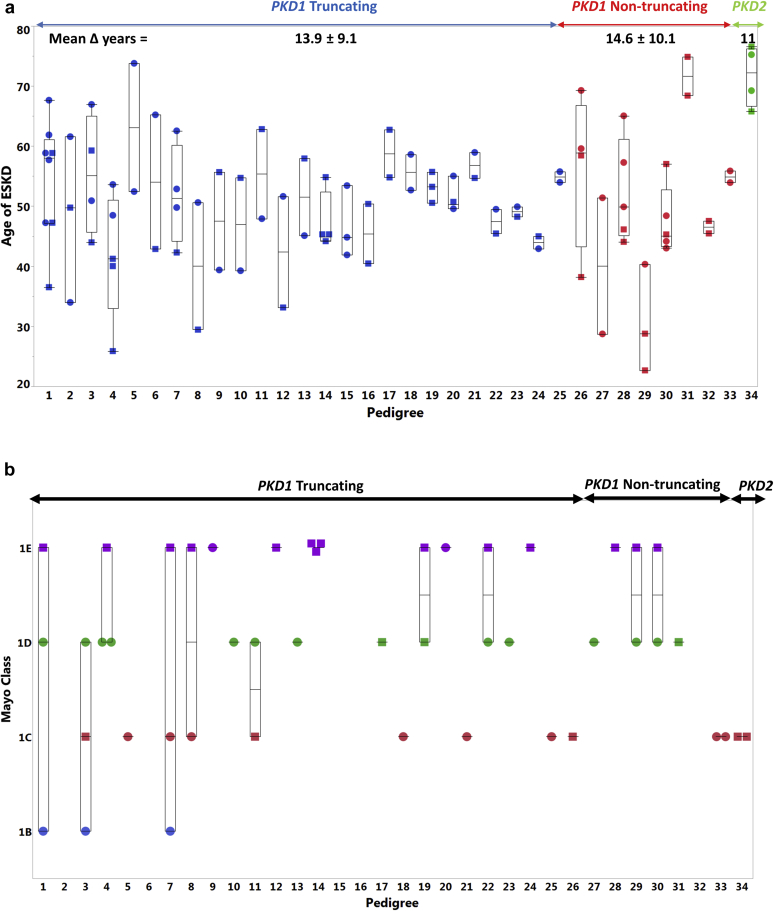
(a) Age of end-stage kidney disease (ESKD) is plotted for each pedigree based on their familial PKD mutation. The difference in ESKD age onset between the youngest and oldest family member was calculated. The mean and SD of the age differences is noted. (b) Mayo Image Class (MIC) is plotted for each pedigree based on their familial PKD mutation.

To ascertain the intrafamilial variability of disease severity, we plotted the age at ESKD and MIC for each pedigree. We included all patients who had genetic testing and had at least 1 family member who also reached ESKD (*n* = 98). A high variability in age of ESKD onset was noted among patients sharing the same PKD mutation types (Figure 4a). In addition, the family members with available imaging had variable MIC at time of ESKD (*n* = 47) (Figure 4b). To further illustrate the variability of kidney volumes at ESKD within each pedigree, we plotted HtTKV over age when family members reached ESKD (Supplementary Figure S4).

## Discussion

The ADPKD phenotype is heterogeneous, as highlighted by our cohort with a range of ESKD age of onset from 23 to 87 years old. Understanding the characteristics associated with age of ESKD onset is critical, given its implication on modifying the disease course. Despite the phenotypic variability, observational studies of large ADPKD cohorts, including CRISP^5^, ^6^, ^7^, ^8^ and pooled registry datasets,^24^^,^^25^ have shown that HtTKV strongly predicts the risk of GFR decline and progression to ESKD. The number of patients who reached ESKD in these cohorts was relatively low at the time of analysis (42 [18.6%] and 88 [5.4%]). In this study, we uniquely provide granular clinical, radiological and genetic data on a large cohort of patients with ADPKD who reached ESKD.

Contrary to our expectations, we found that HtTKV at time of ESKD varied substantially by age at ESKD in our cohort. Patients who reached ESKD at older ages had smaller HtTKV as compared with those who reached ESKD at young ages. HtTKV was smaller by an average of 12.3% at time of ESKD with each decade of life. This novel finding provides insight on the possible underlying mechanism leading to ESKD in ADPKD, which likely differs between younger and older individuals.

Although a negative correlation between age and HtTKV at time of ESKD was observed in both women and men overall, sex appeared to have a marked effect on this relationship, as these findings were statistically significant in women but were attenuated in men. Most patients with ESKD before 47 years of age were men, whereas a larger percentage of patients with ESKD older than 61 years were women. When patients were stratified according to disease severity reflected by the MIC, those with the most severe disease (MIC 1E patients) had fully penetrant PKD mutations (92%) and were predominately men (70%). This suggests that PKD mutation strength and male gender are major determinants of rapid cystic expansion and is consistent with the results of the CRISP study^5^^,^^6^ and the aggravating effect of testosterone in several rodent models of PKD.^26^, ^27^, ^28^, ^29^ In contrast, most patients with ESKD with class MIC 1B or 2B were women (82%) with fewer fully penetrant PKD mutations (55%). This suggests that the slower cystic expansion in these patients allowed other age-related factors to play a more important role in the decline of kidney function. We moreover studied whether the PKD mutation strength and various age-related risk factors for cardiovascular diseases were associated with HtTKV at ESKD in these patients. We found that high Framingham scores and weak PKD mutations in men, and high Framingham and macrovascular disease scores in women, were associated with lower HtTKV at ESKD in the univariate analysis. Only macrovascular disease scores in women were associated with lower HtTKV at ESKD in the multivariate analysis when age was included in the model. This association does not prove a causal relationship between macrovascular disease and reaching ESKD in women. The higher percentage of women with ESKD in the older age groups compared with the group <47 years of age may be due in part to slower disease progression, possibly owing to the effect of estrogens on the renal cystic disease. In experimental PKD models, ovariectomy attenuated the protective effect of female gender and estrogens slowed disease progression.^30^^,^^31^ Menopause may affect PKD progression, similar to the known effect of menopause on cardiovascular disease progression. In addition, prevalence rates of hypertension and cardiovascular disease are lower in women until the sixth decade of life and then increase exponentially matching or exceeding those observed in men.^32^^,^^33^ The reasons for this phenomenon are thought to be related to changes in sex hormones and differences in vascular aging, including endothelial dysfunction and large elastic artery stiffening.^34^, ^35^, ^36^ Metabolic syndrome and arterial stiffness associated with type 2 diabetes are more pronounced in aging women than men.^34^, ^35^, ^36^ In aging women with type 2 diabetes, the relative risk of cardiovascular disease, ischemic heart disease, and stroke is higher than in similarly affected aging men even after differences in other major cardiovascular risk factors have been considered.^37^, ^38^, ^39^, ^40^ Therefore, it is possible but not proven that the steeper negative slope of HtTKV at ESKD observed in women compared with men with ADPKD may be in part due to the accelerated vascular aging observed in older women. Whether there is causal association between accelerated vascular aging and ADPKD progression in women after menopause deserves further study. If there was, estrogen replacement therapy should not be avoided in menopausal women with ADPKD, unless there is evidence of moderate to severe concomitant polycystic liver disease.^41^, ^42^, ^43^

**Figure 5:**
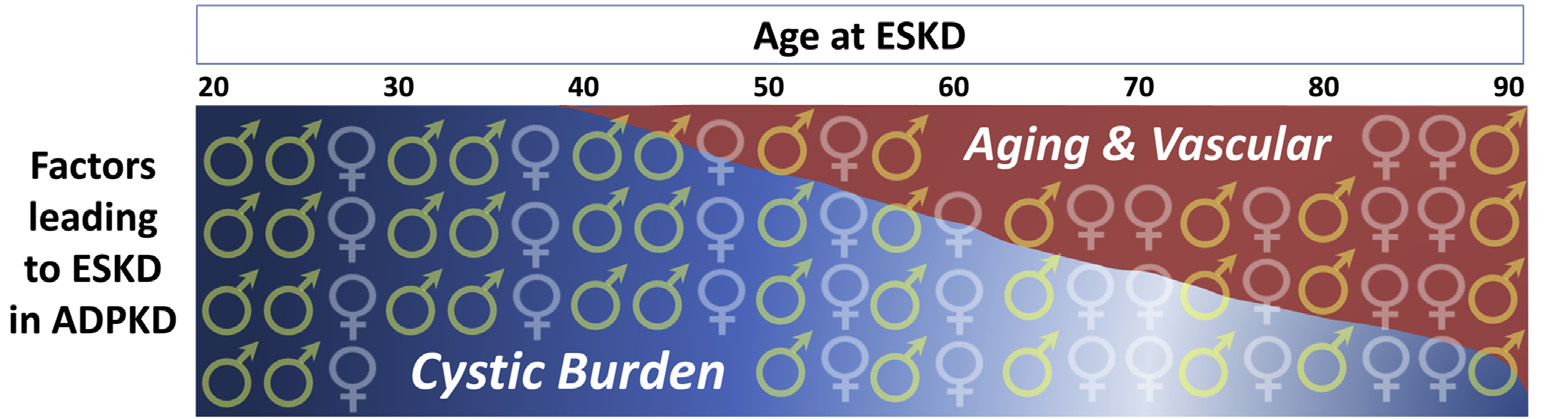
Graphic summarizing the factors contributing to renal function decline and emergence of end-stage kidney disease (ESKD) in autosomal dominant polycystic kidney disease (ADPKD). Cystic growth is the predominant mechanism in younger patients who progress earlier and faster to ESKD, whereas aging-related factors, likely vascular, contribute substantially as patients age. Aging-related vascular factors associated with hormonal changes may contribute to progression of the disease in women following menopause.

The finding that kidney volumes at ESKD are smaller with advancing age, particularly in women, emphasizes the complexity of mechanisms contributing to disease progression in ADPKD and how they may be influenced by age and differ between younger and older individuals. Cystic growth is the predominant mechanism in younger patients who progress earlier and faster to ESKD, whereas aging-related factors likely vascular in nature contribute substantially as patients age (Figure 5). In addition to cystic expansion, extensive vascular remodeling has been proposed to play an important role in the progression of the disease.^44^, ^45^, ^46^ The polycystins are expressed in endothelial cells,^47^^,^^48^ and endothelial cell dysfunction may occur early in ADPKD.^49^, ^50^, ^51^ The polycystins are also expressed in the vascular smooth muscle^52^, ^53^, ^54^ and may play a role in sensing the mechanical environment of the vascular wall.^55^, ^56^, ^57^ A reduction in renal blood flow precedes the development of hypertension^58^ and precedes and predicts the decline in GFR.^15^^,^^59^ The administration of an angiotensin-converting enzyme inhibitor partially reverses the reduction in renal blood flow.^60^^,^^61^ Remodeling of the renal vasculature has been reported to occur at early stages in rodent models of the disease.^62^^,^^63^ Therefore, it is possible that renal vascular remodeling, independent from cyst expansion, may contribute to the renal functional decline in ADPKD and that its contribution becomes more relevant in patients with slowly progressive ADPKD.

The effect of aging on kidney structure and function is not unique to but may be exaggerated in patients with ADPKD. Aging of noncystic kidneys is associated with micro-anatomic (arteriosclerosis, glomerulosclerosis, tubular atrophy with interstitial fibrosis, decreased number of glomeruli, and to some extent compensatory hypertrophy of remaining nephrons) and macro-anatomic (smaller cortical volume and TKV and renal cysts) structural changes.^64^^,^^65^ After age 50, TKV starts declining and GFR declines at a rate of 6.3 ml/min per 1.73 m^2^ per decade.^66^ A reduced nephron endowment at birth is also thought to predispose to CKD later in life.^67^ Patients with ADPKD with a low birth weight, usually associated with a reduced nephron endowment, reach ESKD at an earlier age.^68^ There has been an increased interest in understanding the molecular biology of kidney aging. Mitochondrial oxidative stress and damage may be a major contributor.^69^ Interestingly, although the polycystins affect mitochondrial metabolism,^70^^,^^71^ mitochondrial dysfunction and oxidative stress likely play a role in the pathogenesis of ADPKD.^72^^,^^73^ Therefore, it seems likely that polycystic kidneys are particularly susceptible to aging.

An intriguing observation of this study is the large variability of disease severity and ages at ESKD within the same family. This finding highlights the complexity of the factors, beyond PKD mutations, involved in determining the severity of ADPKD. Our study is in alignment with the recent reporting that showed the presence of extreme kidney disease discordance in at least 12% of families with ADPKD, regardless of the underlying mutated gene or mutation class.^74^

One of the major strengths of this study is the size of the cohort and granularity and depth of individual patient chart review. To our knowledge, this is the first study analyzing HtTKV measurements at time of ESKD, revealing implications for clinical practice. Although disease-modifying therapies targeting epithelial cell proliferation and fluid secretion are particularly important for patients with rapidly progressive disease, therapies targeting endothelial cell dysfunction and vascular remodeling may be important not only for patients with rapidly progressive disease but particularly for less rapidly progressive disease. The slow progression of cystic disease in older patients with ADPKD along with the greater influence of other factors such as vascular remodeling may be the reason why a beneficial effect of tolvaptan, a drug that blunts cAMP-dependent cell proliferation and fluid secretion, could not be demonstrated over 1 year of follow-up in patients with ADPKD older than 55 years and an estimated GFR of 25 to 44 ml per minute per 1.73 m^2^ in the REPRISE clinical trial.^75^

There are several limitations to our study, given the retrospective nature of the study design. First, our study might have underestimated macrovascular disease, as some of the patients had only MRI abdominal images, which did not allow an assessment of the abdominal aortic calcification and not all patients had a comprehensive cardiac testing. It is also possible that some patients died from macrovascular disease before reaching ESKD, precluding them from being included in this study. However, only 0.7% of the patients died before reaching to ESKD, indicating that the selection bias in this study is minimal. Genetic analysis was not available for the entire cohort. Most patients in this cohort are white, therefore the generalizability of this study’s results to other ethnic and racial groups may be limited. Although a referral bias could be present given that Mayo Clinic is a tertiary center, almost two-thirds of the patients are from Minnesota and surrounding states. The cohort is representative of the general ADPKD population except for the race limitation. In addition, MIC may change over time. However, MIC remained stable in most patients over time in both the Mayo and CRISP cohort with only 11% to 22% of patients progressing to an immediate higher class.^14^ Last, the accuracy of the TKV measurement at ESKD could be affected, as the imaging studies were obtained over an interval of 2.25 years around the time of onset of ESKD. However, the vast majority of patients had their imaging performed within a year of onset of ESKD, with median time of the imaging being 4.8 months before ESKD (interquartile range 0.7–11 months).

In conclusion, the kidney volume at ESKD is smaller with advancing age in patients with ADPKD, particularly in women. This novel finding provides insight into the possible underlying mechanisms leading to ESKD in ADPKD, which likely differ between younger and older individuals. Cystic growth is the predominant mechanism in younger patients who progress earlier and faster to ESKD, whereas other aging-related factors, likely vascular, contribute substantially as patients age. Aging-related vascular factors associated with hormonal changes may contribute to progression of the disease in women following menopause.

## Disclosures

PCH reports receiving grants and/or research reagents from Amgen, Inc., Bayer AG, Genzyme Corporation, GlaxoSmithKline (GSK), Mitobridge Inc., Otsuka Pharmaceuticals, Palladio Biosciences, Regulus Therapeutics, and Vertex Pharmaceuticals, all outside the submitted work. PCH also reports a position on the Clinical Advisory Board of Mironid, honoraria from Otsuka Pharmaceuticals and Vertex Pharmaceuticals, and other fees from Otsuka Pharmaceuticals. VET reports grants and/or other fees from Blueprint Medicines, Mironid, Otsuka Pharmaceuticals, Palladio Biosciences, Sanofi Genzyme, and Regulus Therapeutics, all outside the submitted work. All the other authors declared no competing interests.

## Supplementary Material

Supplementary File (PDF)

**Table S1.** Comparison of demographic, clinical, and genotypic characteristics of patients at ESKD included in this study versus those excluded.

**Table S2.** Characteristics of patients reaching kidney failure by age 40.

**Table S3.** Association between patient characteristics and log10 (HtTKV) at ESKD with genetic testing (sex-specific univariate analysis).

**Table S4.** Association between patient characteristics and log10 (HtTKV) at ESKD reached between 2010 and 2018 (sex-specific univariate analysis).

**Figure S1.** Breakdown of patients with ADPKD who reached ESKD stratified by sex and age group (5-year interval).

**Figure S2.** The Log10 HtTKV at ESKD was plotted against the age at ESKD and divided by 3 periods (1992–2000, 2001–2009, and 2010–2018). Best-fit lines and regression slopes are determined for all patients and by sex. The regression slope represents the percent change in HtTKV per decade of age at time of ESKD.

**Figure S3.** The Log10 HtTKV at ESKD was plotted against the age at ESKD and stratified by patients who were receiving dialysis (or reached CKD stage 5) and patients who received preemptive kidney transplantation. The regression slope represents the percent change in HtTKV per decade of age at time of ESKD.

**Figure S4.** HtTKV at ESKD is plotted for each family member over age of ESKD. Each dot, representing a patient, is annotated with the pedigree’s number.

**STROBE Checklist**

## References

[1] Chebib F.T., Torres V.E. (2016). Autosomal dominant polycystic kidney disease: core curriculum. 2016. Am J Kidney Dis.

[2] Hateboer N., v Dijk M.A., Bogdanova N. (1999). Comparison of phenotypes of polycystic kidney disease types 1 and 2. European PKD1-PKD2 Study Group. Lancet.

[3] Cornec-Le Gall E., Audrezet M.P., Chen J.M. (2013). Type of PKD1 mutation influences renal outcome in ADPKD. J Am Soc Nephrol.

[4] Heyer C.M., Sundsbak J.L., Abebe K.Z. (2016). Predicted mutation strength of nontruncating PKD1 mutations aids genotype-phenotype correlations in autosomal dominant polycystic kidney disease. J Am Soc Nephrol.

[5] Lavu S., Vaughan L.E., Senum S.R. (2020). The value of genotypic and imaging information to predict functional and structural outcomes in ADPKD. JCI Insight.

[6] Grantham J.J., Torres V.E., Chapman A.B. (2006). Volume progression in polycystic kidney disease. N Engl J Med.

[7] Chapman A.B., Bost J.E., Torres V.E. (2012). Kidney volume and functional outcomes in autosomal dominant polycystic kidney disease. Clin J Am Soc Nephrol.

[8] Yu A.S.L., Shen C., Landsittel D.P. (2018). Baseline total kidney volume and the rate of kidney growth are associated with chronic kidney disease progression in Autosomal Dominant Polycystic Kidney Disease. Kidney Int.

[9] Harris P.C., Bae K.T., Rossetti S. (2006). Cyst number but not the rate of cystic growth is associated with the mutated gene in autosomal dominant polycystic kidney disease. J Am Soc Nephrol.

[10] Casteleijn N.F., Blais J.D., Chapman A.B. (2017). Tolvaptan and kidney pain in patients with autosomal dominant polycystic kidney disease: secondary analysis from a randomized controlled trial. Am J Kidney Dis.

[11] Irazabal M.V., Abebe K.Z., Bae K.T. (2017). Prognostic enrichment design in clinical trials for autosomal dominant polycystic kidney disease:the HALT-PKD clinical trial. Nephrol Dial Transplant.

[12] US Food and Drug Administration Center for Drug Evaluation and Research: Qualification of biomarker total kidney volume in studies for treatment of autosomal dominant polycystic kidney disease guidance for industry. https://www.fda.gov/regulatory-information/search-fda-guidance-documents/qualification-biomarker-total-kidney-volume-studies-treatment-autosomal-dominant-polycystic-kidney.

[13] European Medicines Agency Qualification opinion. Total Kidney Volume (TKV) as a prognostic biomarker for use in clinical trials evaluating patients with Autosomal Dominant Polycystic Kidney Disease (ADPKD). http://www.ema.europa.eu/docs/en_GB/document_library/Regulatory_and_procedural_guideline/2015/11/WC500196569.pdf.

[14] Irazabal M.V., Rangel L.J., Bergstralh E.J. (2015). Imaging classification of autosomal dominant polycystic kidney disease: a simple model for selecting patients for clinical trials. J Am Soc Nephrol.

[15] Torres V.E., King B.F., Chapman A.B. (2007). Magnetic resonance measurements of renal blood flow and disease progression in autosomal dominant polycystic kidney disease. Clin J Am Soc Nephrol.

[16] Grantham J.J., Torres V.E. (2016). The importance of total kidney volume in evaluating progression of polycystic kidney disease. Nat Rev Nephrol.

[17] Wilson P.W., D'Agostino R.B., Levy D. (1998). Prediction of coronary heart disease using risk factor categories. Circulation.

[18] Levey A.S., Stevens L.A., Schmid C.H. (2009). A new equation to estimate glomerular filtration rate. Ann Intern Med.

[19] Cornec-Le Gall E., Olson R.J., Besse W. (2018). Monoallelic mutations to DNAJB11 cause atypical autosomal-dominant polycystic kidney disease. Am J Hum Genet.

[20] Rossetti S., Consugar M.B., Chapman A.B. (2007). Comprehensive molecular diagnostics in autosomal dominant polycystic kidney disease. J Am Soc Nephrol.

[21] Rossetti S., Kubly V.J., Consugar M.B. (2009). Incompletely penetrant PKD1 alleles suggest a role for gene dosage in cyst initiation in polycystic kidney disease. Kidney Int.

[22] Hopp K., Cornec-Le Gall E., Senum S.R. (2020). Detection and characterization of mosaicism in autosomal dominant polycystic kidney disease. Kidney Int.

[23] Chebib F.T., Perrone R.D., Chapman A.B. (2018). A practical guide for treatment of rapidly progressive ADPKD with tolvaptan. J Am Soc Nephrol.

[24] Perrone R.D., Mouksassi M.S., Romero K. (2017). Total kidney volume is a prognostic biomarker of renal function decline and progression to end-stage renal disease in patients with autosomal dominant polycystic kidney disease. Kidney Int Rep.

[25] Perrone R.D., Mouksassi M.S., Romero K. (2017). A drug development tool for trial enrichment in patients with autosomal dominant polycystic kidney disease. Kidney Int Rep.

[26] Smith L.A., Bukanov N.O., Husson H. (2006). Development of polycystic kidney disease in juvenile cystic kidney mice:insights into pathogenesis, ciliary abnormalities, and common features with human disease. J Am Soc Nephrol.

[27] Nagao S., Kusaka M., Nishii K. (2005). Androgen receptor pathway in rats with autosomal dominant polycystic kidney disease. J Am Soc Nephrol.

[28] Sandhu S., Silbiger S.R., Lei J. (1997). Effects of sex hormones on fluid and solute transport in Madin-Darby canine kidney cells. Kidney Int.

[29] Cowley B.D., Rupp J.C., Muessel M.J. (1997). Gender and the effect of gonadal hormones on the progression of inherited polycystic kidney disease in rats. Am J Kidney Dis.

[30] Stringer K.D., Komers R., Osman S.A. (2005). Gender hormones and the progression of experimental polycystic kidney disease. Kidney Int.

[31] Anderson S., Oyama T.T., Lindsley J.N. (2012). 2-Hydroxyestradiol slows progression of experimental polycystic kidney disease. Am J Physiol Renal Physiol.

[32] Yoon S.S., Carroll M.D., Fryar C.D. (2015). Hypertension prevalence and control among adults: United States, 2011–2014. NCHS Data Brief.

[33] Benjamin E.J., Muntner P., Alonso A. (2019). Heart disease and stroke statistics-2019 update: a report from the American Heart Association. Circulation.

[34] Russo C., Jin Z., Palmieri V. (2012). Arterial stiffness and wave reflection:sex differences and relationship with left ventricular diastolic function. Hypertension.

[35] Coutinho T., Borlaug B.A., Pellikka P.A. (2013). Sex differences in arterial stiffness and ventricular-arterial interactions. J Am Coll Cardiol.

[36] Ogola B.O., Zimmerman M.A., Clark G.L. (2018). New insights into arterial stiffening: does sex matter?. Am J Physiol Heart Circ Physiol.

[37] Regensteiner J.G., Golden S., Huebschmann A.G. (2015). Sex differences in the cardiovascular consequences of diabetes mellitus: a scientific statement from the American Heart Association. Circulation.

[38] Rodriguez-Campello A., Jimenez-Conde J., Ois A. (2017). Sex-related differences in abdominal obesity impact on ischemic stroke risk. Eur J Neurol.

[39] Li X., Li X., Lin H. (2017). Metabolic syndrome and stroke: a meta-analysis of prospective cohort studies. J Clin Neurosci.

[40] Madonna R., Balistreri C.R., De Rosa S. (2019). Impact of sex differences and diabetes on coronary atherosclerosis and ischemic heart disease. J Clin Med.

[41] Sherstha R., McKinley C., Russ P. (1997). Postmenopausal estrogen therapy selectively stimulates hepatic enlargement in women with autosomal dominant polycystic kidney disease. Hepatology.

[42] Alvaro D., Onori P., Alpini G. (2008). Morphological and functional features of hepatic cyst epithelium in autosomal dominant polycystic kidney disease. Am J Pathol.

[43] Chebib F.T., Jung Y., Heyer C.M. (2016). Effect of genotype on the severity and volume progression of polycystic liver disease in autosomal dominant polycystic kidney disease. Nephrol Dial Transplant.

[44] Ritter R., Siafarikas K. (1976). Hemihypertrophy in a boy with renal polycystic disease:varied patterns of presentation of renal polycystic disease in his family. Pediatr Radiol.

[45] Schaft F. (1931). Hypertension in cases of congenital polycystic kidney. Arch Intern Med.

[46] Schaft F. (1930). Hypertension and vascular studies in congenital polycystic kidney [thesis].

[47] Nauli S.M., Kawanabe Y., Kaminski J.J. (2008). Endothelial cilia are fluid shear sensors that regulate calcium signaling and nitric oxide production through polycystin-1. Circulation.

[48] AbouAlaiwi W.A., Takahashi M., Mell B.R. (2009). Ciliary polycystin-2 is a mechanosensitive calcium channel involved in nitric oxide signaling cascades. Circ Res.

[49] Peterson K.M., Franchi F., Loeffler D.L. (2013). Endothelial dysfunction occurs prior to clinical evidence of polycystic kidney disease. Am J Nephrol.

[50] Klawitter J., Reed-Gitomer B.Y., McFann K. (2014). Endothelial dysfunction and oxidative stress in polycystic kidney disease. Am J Physiol Renal Physiol.

[51] Nowak K.L., Wang W., Farmer-Bailey H. (2018). Vascular dysfunction, oxidative stress, and inflammation in autosomal dominant polycystic kidney disease. Clin J Am Soc Nephrol.

[52] Griffin M.D., Torres V.E., Grande J.P. (1997). Vascular expression of polycystin. J Am Soc Nephrol.

[53] Torres V.E., Cai Y., Chen X. (2001). Vascular expression of polycystin-2. J Am Soc Nephrol.

[54] Qian Q., Li M., Cai Y. (2003). Analysis of the polycystins in aortic vascular smooth muscle cells. J Am Soc Nephrol.

[55] Sharif-Naeini R., Folgering J.H., Bichet D. (2009). Polycystin-1 and -2 dosage regulates pressure sensing. Cell.

[56] Patel A., Honore E. (2010). Polycystins and renovascular mechanosensory transduction. Nat Rev Nephrol.

[57] Retailleau K., Duprat F. (2014). Polycystins and partners: proposed role in mechanosensitivity. J Physiol.

[58] Barrett B.J., Foley R., Morgan J. (1994). Differences in hormonal and renal vascular responses between normotensive patients with autosomal dominant polycystic kidney disease and unaffected family members. Kidney Int.

[59] King B.F., Torres V.E., Brummer M.E. (2003). Magnetic resonance measurements of renal blood flow as a marker of disease severity in autosomal-dominant polycystic kidney disease. Kidney Int.

[60] Chapman A.B., Johnson A., Gabow P.A. (1990). The renin-angiotensin-aldosterone system and autosomal dominant polycystic kidney disease. N Engl J Med.

[61] Torres V.E., Wilson D.M., Burnett J.C. (1991). Effect of inhibition of converting enzyme on renal hemodynamics and sodium management in polycystic kidney disease. Mayo Clin Proc.

[62] Ogunlade O., Connell J.J., Huang J.L. (2018). In vivo three-dimensional photoacoustic imaging of the renal vasculature in preclinical rodent models. Am J Physiol Renal Physiol.

[63] Xu R., Franchi F., Miller B. (2013). Polycystic kidneys have decreased vascular density: a micro-CT study. Microcirculation.

[64] Denic A., Glassock R.J., Rule A.D. (2016). Structural and functional changes with the aging kidney. Adv Chronic Kidney Dis.

[65] Wang M., Avula B., Wang Y.H. (2014). An integrated approach utilising chemometrics and GC/MS for classification of chamomile flowers, essential oils and commercial products. Food Chem.

[66] Rule A.D., Amer H., Cornell L.D. (2010). The association between age and nephrosclerosis on renal biopsy among healthy adults. Ann Intern Med.

[67] Vikse B.E., Irgens L.M., Leivestad T. (2008). Low birth weight increases risk for end-stage renal disease. J Am Soc Nephrol.

[68] Orskov B., Christensen K.B., Feldt-Rasmussen B. (2012). Low birth weight is associated with earlier onset of end-stage renal disease in Danish patients with autosomal dominant polycystic kidney disease. Kidney Int.

[69] Dai D.F., Chiao Y.A., Marcinek D.J. (2014). Mitochondrial oxidative stress in aging and healthspan. Longev Healthspan.

[70] Menezes L.F., Germino G.G. (2019). The pathobiology of polycystic kidney disease from a metabolic viewpoint. Nat Rev Nephrol.

[71] Kuo I.Y., Brill A.L., Lemos F.O. (2019). Polycystin 2 regulates mitochondrial Ca(2+) signaling, bioenergetics, and dynamics through mitofusin 2. Sci Signal.

[72] Padovano V., Podrini C., Boletta A. (2018). Metabolism and mitochondria in polycystic kidney disease research and therapy. Nat Rev Nephrol.

[73] Hajarnis S., Lakhia R., Yheskel M. (2017). microRNA-17 family promotes polycystic kidney disease progression through modulation of mitochondrial metabolism. Nat Commun.

[74] Lanktree M.B., Guiard E., Li W. (2019). Intrafamilial variability of ADPKD. Kidney Int Rep.

[75] Torres V.E., Chapman A.B., Devuyst O. (2017). Tolvaptan in later-stage autosomal dominant polycystic kidney disease. N Engl J Med.

