# Supplementary material for: Characteristics of Patients with End-Stage Kidney Disease in ADPKD
Source: Kidney Int Rep. 2020 Dec 31;6(3):755–67. doi: 10.1016/j.ekir.2020.12.016 (PMC7938061; doi:10.1016/j.ekir.2020.12.016)
Supplement: Supplementary File (PDF) [file mmc1.pdf]

Supplemental Table of Contents

Table S1: Comparison of demographic, clinical, and genotypic characteristics of patients at ESKD included in this study versus those excluded.

Table S2: Characteristics of patients reaching kidney failure by age 40.

Table S3: Association between patient characteristics and log10 (HtTKV) at ESKD with genetic testing (sex-specific univariate analysis).

Table S4: Association between patient characteristics and log10 (HtTKV) at ESKD reached between 2010 and 2018 (sex-specific univariate analysis).

Figure S1: Breakdown of patients with ADPKD who reached ESKD stratified by sex and age group (5 years interval).

Figure S2: The Log10 HtTKV at ESKD was plotted against the age at ESKD and divided by three periods (1992-2000, 2001-2009, and 2010-2018). Best-fit lines and regression slopes are determined for all patients and by sex. The regression slope represents the percent change in HtTKV per decade of age at time of ESKD.

Figure S3: The Log10 HtTKV at ESKD was plotted against the age at ESKD and stratified by patients who were receiving dialysis (or reached CKD stage 5) and patients who received preemptive kidney transplantation. The regression slope represents the percent change in HtTKV per decade of age at time of ESKD.

Figure S4: HtTKV at ESKD is plotted for each family member over age of ESKD. Each dot, representing a patient, is annotated with the pedigree's number.

STROBE Checklist

Table S1: Comparison of demographic, clinical, and genotypic characteristics of patients at ESKD included in this study versus those excluded.

	All N=1076		Included N=290		Excluded N=786	
**Patient Characteristic**	**N**	**mean ± SD or n (%)**	**N**	**mean ± SD or n (%)**	**N**	**mean ± SD or n (%)**
**Male sex**	1076	560 (52%)	290	138 (48%)	786	422 (54%)
**Caucasian**	1076	970 (90%)	290	268 (92%)	786	702 (89%)
**Age at ESKD, years**	1076	54.7 ± 12.3	290	54.5 ± 11.3	786	54.7 ± 12.6
**eGFR, ml/min/1.73m²**						
All	1076	11.9 ± 4.9	290	13.0 ± 4.6	786	10.4 ± 4.2
Preemptive renal transplant	465	13.5 ± 4.5	179	14.1 ± 4.6	287	11.7 ± 3.8
Dialysis	435	10.8 ± 4.8	80	10.8 ± 4.6	355	10.9 ± 5.0
CKD stage 5	175	10.2 ± 3.6	31	12.1 ± 1.8	144	9.8 ± 3.8
**Genotype**	320		182		138	
PKD1 Truncating		194 (61%)		110 (60%)		84 (61%)
PKD1 Non-truncating		100 (31%)		55 (30%)		45 (33%)
PKD2		26 (8%)		17 (10%)		9 (6%)
**BMI, kg/m²**	1076	32.2 ± 9.2	290	28.6 ± 5.7	786	34 ± 10

Table S2: Characteristics of patients reaching kidney failure by age 40.

Patient Characteristic	Patients reaching kidney failure by age 40 (N=23)
**Caucasian, %y**	22 (96%)
**Male sex, %y**	11 (48%)
**Age at ESKD (years), mean ± SD**	34.5 ± 4.4
**eGFR (ml/min/1.73m^2), mean ± SD**	
All	14.3 ± 4.8
**HtTKV (mL/m), median (IQR)**	1979 (1320-2850)
**Mayo Image Class, N**	
1D	N=5
1E	N=18
**Severe Class (1C- 1E), %y**	100%
**Genotype, N (%)**	
$PKD1^{T}$	14/17(82%)
$PKD1^{NT1}$	2/17 (12%)
No mutation detected	1/ 17 (6%)
**BMI (kg/m^2), mean ± SD**	30.7 ± 6.9
**History of hypertension, %y**	22 (95%)
**History of Smoking, %y**	8 (35%)
**History of Dyslipidemia, %y**	5 (22%)
**Macrovascular Disease, %y**	1 (4%)
**Diabetes Mellitus, %y**	0 (0%)
**Persistent hematuria, %y**	10 (44%)
**Tuberous sclerosis**	1 (4%)
**Possible NSAIDs use**	3 (13%)
**Framingham score characteristics**	**N=18**
Framingham Score (%), mean ± SD	4.3 ± 2.1
High risk Framingham score (≥20%), %y	0 (0%)

Table S3 – Association between patient characteristics and log10 (HtTKV) at ESKD with genetic testing (sex-specific univariate analysis).

Predictor	N	Estimate*	95% CI	% change HtTKV **	p-value
**2A - Univariate analysis- Male patients**					
**Age at ESKD (per decade)**	80	-0.040	-0.085, 0.004	-8.8%	0.23
**Genotype**					
$PKD1^{T}$-$PKD1^{NT1}$	55	REF	REF	REF	REF
$PKD1^{NT2}$-PKD2	25	-0.131	-0.242, -0.019	-26.0%	**0.02**
**BMI (per 5 Kg/m^2)**	80	0.029	-0.018, 0.077	6.9%	0.23
**History of Smoking**	80	0.058	0.049, 0.165	14.3%	0.28
**History of Dyslipidemia**	80	-0.045	-0.151, 0.062	-9.8%	0.40
**LDL (per 5 mg/dL)**	40	-0.012	-0.022, -0.002	-2.7%	**0.01**
**HDL (per 5 mg/dL)**	37	-0.012	-0.041, 0.016	-2.7%	0.38
**Framingham Score (per 5%)**	57	-0.028	-0.054, -0.003	-6.2%	**0.02**
**High-Risk Score (≥20%)**	57	-0.134	-0.262, -0.007	-26.5%	**0.03**
**Macrovascular Disease**	80	0.091	-0.047, 0.230	23.3%	0.19
**2B- Univariate analysis- Female patients**					
**Age at ESKD (per decade)**	102	-0.068	-0.112, -0.025	-14.5%	**<0.01**
**Genotype**					
$PKD1^{T}$-$PKD1^{NT1}$	84	REF	REF	REF	REF
$PKD1^{NT2}$-PKD2	18	-0.049	-0.172, 0.073	-10.7%	0.42
**BMI (per 5 Kg/m^2)**	101	0.006	-0.032, 0.044	1.4%	0.76
**History of Smoking**	102	0.018	-0.076, 0.114	4.2%	0.69
**History of Dyslipidemia**	100	-0.014	-0.106, 0.078	-3.2%	0.76
**LDL (per 5 mg/dL)**	49	-0.002	-0.013, 0.008	-0.5%	0.61
**HDL (per 5 mg/dL)**	50	-0.015	-0.033, 0.001	-3.4%	0.07
**Framingham Score (per 5%)**	83	-0.024	-0.055, 0.007	-5.4%	0.13
**High-Risk Score (≥20%)**	83	-0.098	-0.253, 0.055	-20.2%	0.21
**Macrovascular Disease**	102	-0.103	-0.224, 0.017	-21.1%	0.09

*Beta coefficient estimates derived from univariate linear regression models using the log base 10 transformation on the outcome (HtTKV)

** Percent change in HtTKV per unit increase in predictor variable was calculated by subtracting 1 from the log of the estimate then multiplying by 100: % change/unit increase in predictor= $(10^{\text{Beta Estimate}} -1)$ x100

Table S4 – Association between patient characteristics and log10 (HtTKV) at ESKD reached between 2010 and 2018 (sex-specific univariate analysis).

Predictor	N	Estimate*	95% CI	% change HtTKV **	p-value
**A - Univariate analysis- Male patients (2010-2018)**					
**Age at ESKD (per decade)**	77	-0.044	-0.086, -0.002	-9.6%	**0.03**
**Genotype**					
$PKD1^{T}$-$PKD1^{NT1}$	34	REF	REF	REF	REF
$PKD1^{NT2}$-PKD2	13	-0.171	-0.325, -0.015	-32.5%	**0.03**
**BMI (per 5 Kg/m^2)**	76	0.025	-0.024, 0.075	5.9%	0.31
**History of Smoking**	77	0.026	-0.083, 0.136	6.2%	0.63
**History of Dyslipidemia**	77	-0.094	-0.204, 0.015	-19.5%	0.09
**LDL (per 5 mg/dL)**	44	-0.008	-0.019, 0.001	-1.8%	0.10
**HDL (per 5 mg/dL)**	44	-0.015	-0.039, 0.008	-3.4%	0.21
**Framingham Score (per 5%)**	50	-0.030	-0.059, -0.002	-6.7%	**0.03**
**High-Risk Score (≥20%)**	50	-0.141	-0.281,-0.002	-27.7%	**0.04**
**Macrovascular Disease**	77	-0.001	-0.128, 0.126	-0.2%	0.98
**B - Univariate analysis- Female patients (2010-2018)**					
**Age at ESKD (per decade)**	89	-0.081	-0.130, -0.031	-17.0%	**<0.01**
**Genotype**					
$PKD1^{T}$-$PKD1^{NT1}$	53	REF	REF	REF	REF
$PKD1^{NT2}$-PKD2	10	0.004	-0.171, 0.179	0.9%	0.96
**BMI (per 5 Kg/m^2)**	87	-0.005	-0.052, 0.041	-1.1%	0.82
**History of Smoking**	89	-0.004	-0.120, 0.110	-0.9%	0.93
**History of Dyslipidemia**	88	-0.010	-0.120, 0.099	2.3%	0.85
**LDL (per 5 mg/dL)**	53	0.004	-0.006, 0.014	0.9%	0.47
**HDL (per 5 mg/dL)**	54	-0.016	-0.034, 0.002	-3.6%	0.09
**Framingham Score (per 5%)**	72	-0.051	-0.085, -0.016	-11.1%	**<0.01**
**High-Risk Score (≥20%)**	72	-0.230	-0.391,-0.068	-41.1%	**<0.01**
**Macrovascular Disease**	89	-0.134	-0.288, 0.020	-26.5%	0.08

*Beta coefficient estimates derived from univariate linear regression models using the log base 10 transformation on the outcome (HtTKV)

** Percent change in HtTKV per unit increase in predictor variable was calculated by subtracting 1 from the log of the estimate then multiplying by 100: % change/unit increase in predictor= $(10^{\text{Beta Estimate}} - 1) \times 100$

Figure S1: Breakdown of patients with ADPKD who reached ESKD stratified by sex and age group (5 years interval).

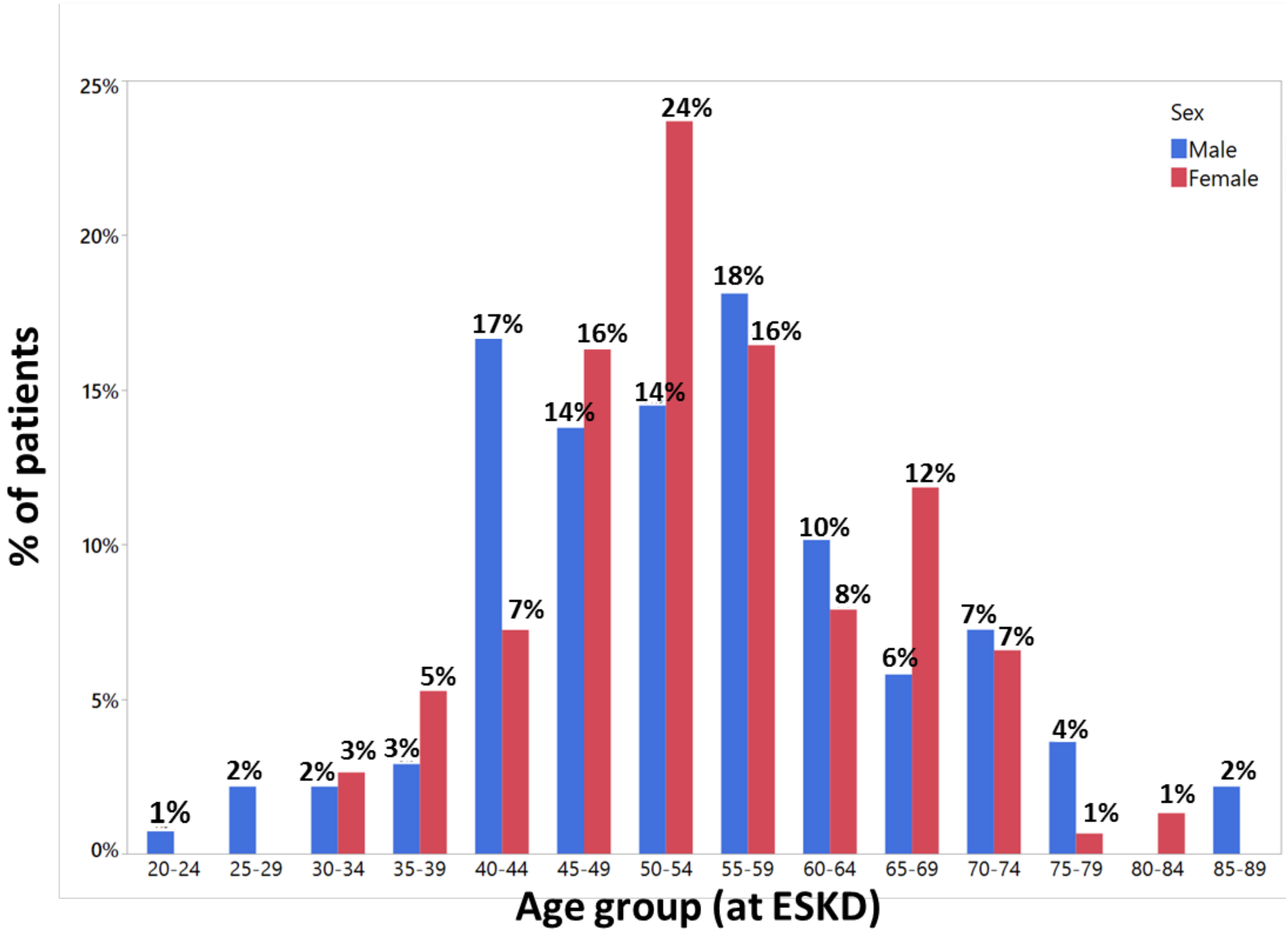

Figure S2: The Log10 HtTKV at ESKD was plotted against the age at ESKD and divided by three periods (1992-2000, 2001-2009, and 2010-2018). Best-fit lines and regression slopes are determined for all patients and by sex. The regression slope represents the percent change in HtTKV per decade of age at time of ESKD.

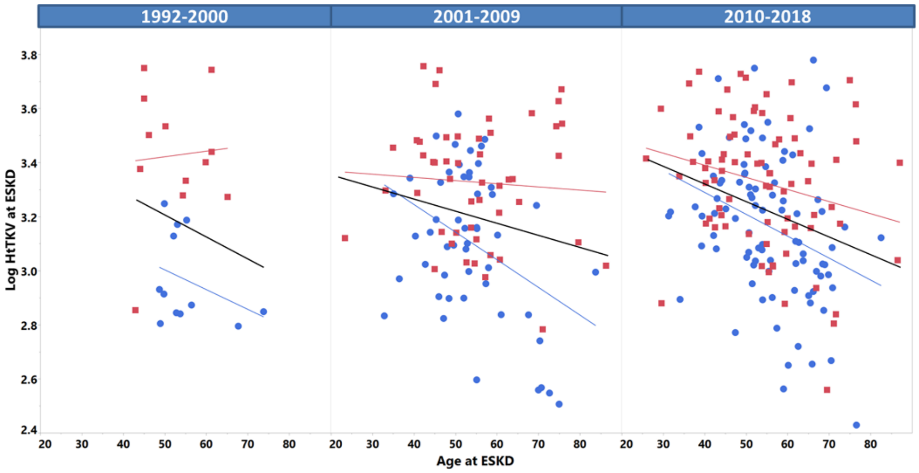

Era	1992-2000		2001-2009		2010-2018	
N, %	24, 8.3%		100, 34.4%		166, 57.3%	
% change of HtTKV per decade	-16.1%<br>p=0.35		-9.2%<br>p=0.06		-13.7%<br>**p<0.01**	
	**Male**	**Female**	**Male**	**Female**	**Male**	**Female**
N	12, 50%	12, 50%	49, 49%	51, 51%	77, 46.4%	89, 53.6%
Mean ESKD age, ± SD	52.5 ± 7.9	55.2 ± 7.8	55.1 ± 12.9	53.8 ± 10.4	53.9 ± 12.8	55.4 ± 10.6
% change of HtTKV per decade	4.9%<br>p=0.83	-15.5%<br>p=0.25	-2.5%<br>p=0.64	-20.9%<br>**p<0.01**	-9.6%<br>**p<=0.03**	-18.15%<br>**p<0.01**

Figure S3: The Log10 HtTKV at ESKD was plotted against the age at ESKD and stratified by patients who were receiving dialysis (or reached CKD stage 5) and patients who received preemptive kidney transplantation. The regression slope represents the percent change in HtTKV per decade of age at time of ESKD.

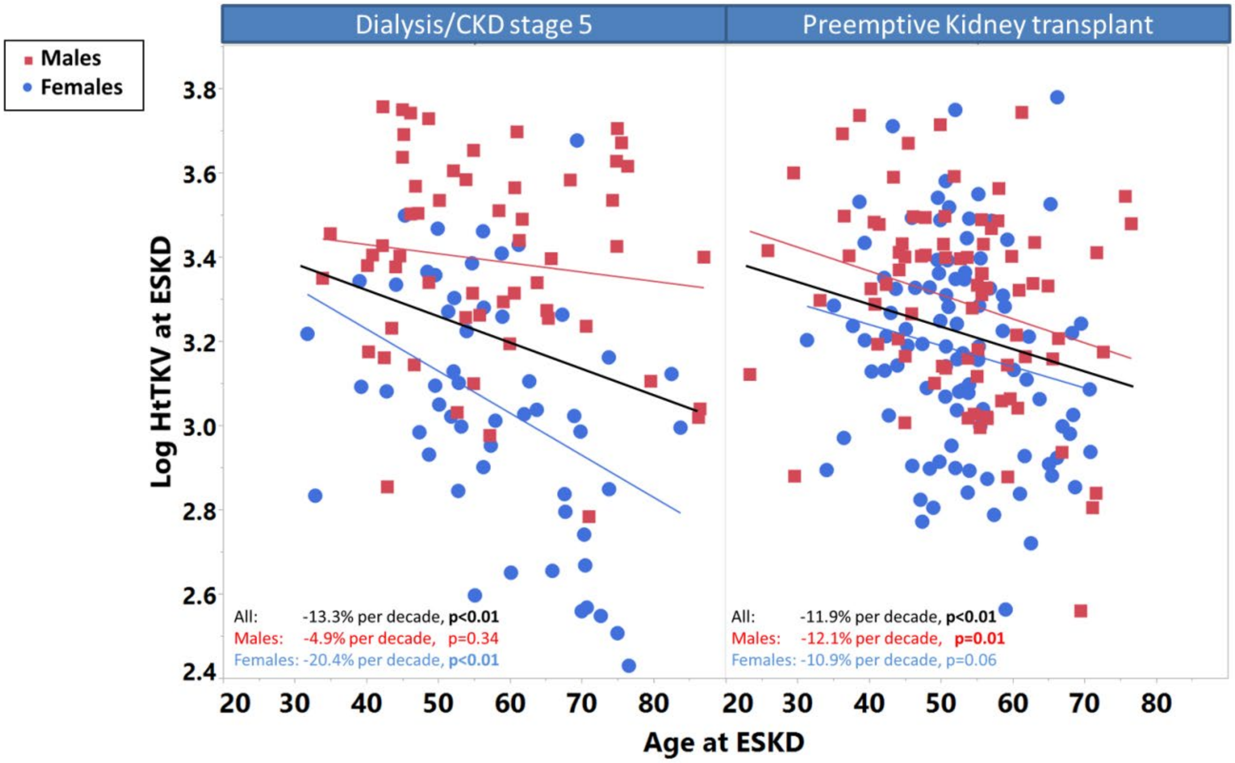

Figure S4: HtTKV at ESKD is plotted for each family member over age of ESKD. Each dot, representing a patient, is annotated with the pedigree's number.

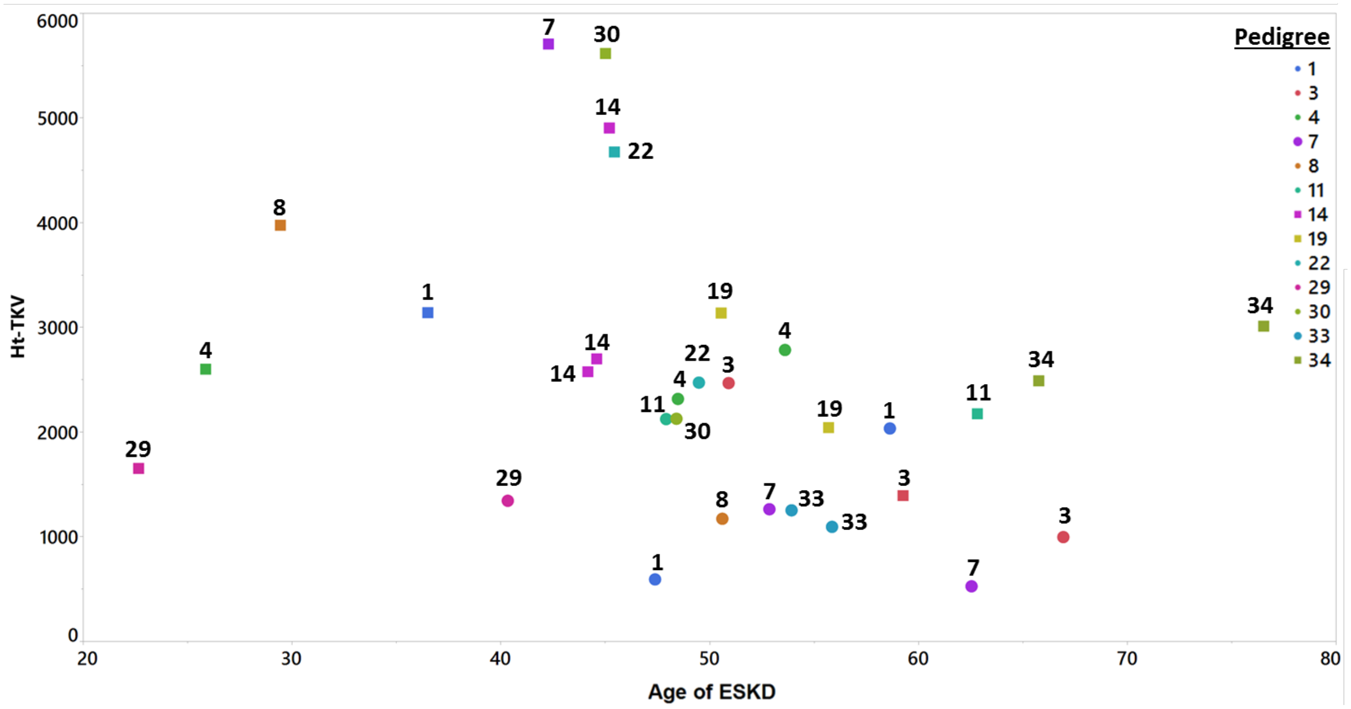

STROBE (Strengthening The Reporting of OBservational Studies in Epidemiology) Checklist

A checklist of items that should be included in reports of observational studies. You must report the page number in your manuscript where you consider each of the items listed in this checklist. If you have not included this information, either revise your manuscript accordingly before submitting or note N/A.

Note: An Explanation and Elaboration article discusses each checklist item and gives methodological background and published examples of transparent reporting. The STROBE checklist is best used in conjunction with this article (freely available on the Web sites of PLoS Medicine at http://www.plosmedicine.org/, Annals of Internal Medicine at http://www.annals.org/, and Epidemiology at http://www.epidem.com/). Information on the STROBE Initiative is available at www.strobe-statement.org.

Section and Item	Item No.	Recommendation	Reported on Page No.
**Title and Abstract**	1	(*a*) Indicate the study's design with a commonly used term in the title or the abstract	2
		(*b*) Provide in the abstract an informative and balanced summary of what was done and what was found	2
**Introduction**			
Background/Rationale	2	Explain the scientific background and rationale for the investigation being reported	3
Objectives	3	State specific objectives, including any prespecified hypotheses	4
**Methods**			
Study Design	4	Present key elements of study design early in the paper	4
Setting	5	Describe the setting, locations, and relevant dates, including periods of recruitment, exposure, follow-up, and data collection	4-8
Participants	6	(*a*) *Cohort study*—Give the eligibility criteria, and the sources and methods of selection of participants. Describe methods of follow-up	-
		Case-control study—Give the eligibility criteria, and the sources and methods of case ascertainment and control selection. Give the rationale for the choice of cases and controls	-
		Cross-sectional study—Give the eligibility criteria, and the sources and methods of selection of participants	4-7
		(*b*) *Cohort study*—For matched studies, give matching criteria and number of exposed and unexposed	NA
		Case-control study—For matched studies, give matching criteria and the number of controls per case	-
Variables	7	Clearly define all outcomes, exposures, predictors, potential confounders, and effect modifiers. Give diagnostic criteria, if applicable	4-12

Section and Item	Item No.	Recommendation	Reported on Page No.
Data Sources/ Measurement	8*	For each variable of interest, give sources of data and details of methods of assessment (measurement). Describe comparability of assessment methods if there is more than one group	4-8
Bias	9	Describe any efforts to address potential sources of bias	10, 16-17
Study Size	10	Explain how the study size was arrived at	8
Quantitative Variables	11	Explain how quantitative variables were handled in the analyses. If applicable, describe which groupings were chosen and why	8
Statistical Methods	12	(*a*) Describe all statistical methods, including those used to control for confounding	7-8
		(*b*) Describe any methods used to examine subgroups and interactions	4-8
		(*c*) Explain how missing data were addressed	5-6
		(*d*) *Cohort study*—If applicable, explain how loss to follow-up was addressed	-
		Case-control study—If applicable, explain how matching of cases and controls was addressed	-
		Cross-sectional study—If applicable, describe analytical methods taking account of sampling strategy	4
		(*e*) Describe any sensitivity analyses	8
**Results**			
Participants	13*	(a) Report numbers of individuals at each stage of study—eg numbers potentially eligible, examined for eligibility, confirmed eligible, included in the study, completing follow-up, and analysed	8
		(b) Give reasons for non-participation at each stage	5-6
		(c) Consider use of a flow diagram	8
Descriptive Data	14*	(a) Give characteristics of study participants (eg demographic, clinical, social) and information on exposures and potential confounders	8-9
		(b) Indicate number of participants with missing data for each variable of interest	4-8
		(c) *Cohort study*—Summarise follow-up time (eg, average and total amount)	-
Outcome Data	15*	*Cohort study*—Report numbers of outcome events or summary measures over time	-
		Case-control study—Report numbers in each exposure category, or summary measures of exposure	-
		Cross-sectional study—Report numbers of outcome events or summary measures	8-12,29

Section and Item	Item No.	Recommendation	Reported on Page No.
Main Results	16	(*a*) Give unadjusted estimates and, if applicable, confounder-adjusted estimates and their precision (eg, 95% confidence interval). Make clear which confounders were adjusted for and why they were included	28-31
		(*b*) Report category boundaries when continuous variables were categorized	-
		(*c*) If relevant, consider translating estimates of relative risk into absolute risk for a meaningful time period	-
Other Analyses	17	Report other analyses done—eg analyses of subgroups and interactions, and sensitivity analyses	8,11
**Discussion**			
Key Results	18	Summarise key results with reference to study objectives	12-18
Limitations	19	Discuss limitations of the study, taking into account sources of potential bias or imprecision. Discuss both direction and magnitude of any potential bias	16-17
Interpretation	20	Give a cautious overall interpretation of results considering objectives, limitations, multiplicity of analyses, results from similar studies, and other relevant evidence	16-17
Generalisability	21	Discuss the generalisability (external validity) of the study results	16-17
**Other Information**			
Funding	22	Give the source of funding and the role of the funders for the present study and, if applicable, for the original study on which the present article is based	19

*Give information separately for cases and controls in case-control studies and, if applicable, for exposed and unexposed groups in cohort and cross-sectional studies.

Once you have completed this checklist, please save a copy and upload it as part of your submission. DO NOT include this checklist as part of the main manuscript document. It must be uploaded as a separate file.
